# Assessment of Potentially Preventable Hospital Readmissions After Major Surgery and Association With Public vs Private Health Insurance and Comorbidities

**DOI:** 10.1001/jamanetworkopen.2021.5503

**Published:** 2021-04-13

**Authors:** Craig S. Brown, John R. Montgomery, Pooja U. Neiman, Glenn K. Wakam, Thomas C. Tsai, Justin B. Dimick, John W. Scott

**Affiliations:** 1 Department of Surgery, University of Michigan, Ann Arbor; 2 Center for Healthcare Outcomes and Policy, University of Michigan, Ann Arbor; 3 Department of Surgery, Brigham and Women’s Hospital, Boston, Massachusetts; 4 National Clinician Scholars Program, University of Michigan, Ann Arbor; 5 Department of Health Policy and Management, Harvard T.H. Chan School of Public Health, Boston, Massachusetts

## Abstract

**Importance:**

Rehospitalization after major surgery is common and represents a significant cost to the health care system. Little is known regarding the causes of these readmissions and the degree to which they may be preventable.

**Objective:**

To evaluate the degree to which readmissions after major surgery are potentially preventable.

**Design, Setting, and Participants:**

This retrospective cohort study used a weighted sample of 1 937 354 patients from the 2017 National Readmissions Database to evaluate all adult inpatient hospitalizations for 1 of 7 common major surgical procedures. Statistical analysis was performed from January 14 to November 30, 2020.

**Main Outcomes and Measures:**

The study calculated 90-day readmission rates as well as rates of readmissions that were considered potentially preventable. Potentially preventable readmissions (PPRs) were defined as those with a primary diagnosis code for superficial surgical site infection, acute kidney injury, aspiration pneumonitis, or any of the Agency for Healthcare Research and Quality–defined ambulatory care sensitive conditions. Multivariable logistic regression was used to identify factors associated with PPRs.

**Results:**

A total weighted sample of 1 937 354 patients (1 048 046 women [54.1%]; mean age, 66.1 years [95% CI, 66.0-66.3 years]) underwent surgical procedures; 164 755 (8.5%) experienced a readmission within 90 days. Potentially preventable readmissions accounted for 29 321 (17.8%) of all 90-day readmissions, for an estimated total cost to the US health care system of approximately $296 million. The most common reasons for PPRs were congestive heart failure exacerbation (34.6%), pneumonia (12.0%), and acute kidney injury (22.5%). In a multivariable model of adults aged 18 to 64 years, patients with public health insurance (Medicare or Medicaid) had more than twice the odds of PPR compared with those with private insurance (adjusted odds ratio, 2.09; 95% CI, 1.94-2.25). Among patients aged 65 years or older, patients with private insured had 18% lower odds of PPR compared with patients with Medicare as the primary payer (adjusted odds ratio, 0.82; 95% CI, 0.74-0.90).

**Conclusions and Relevance:**

This study suggests that nearly 1 in 5 readmissions after surgery are potentially preventable and account for nearly $300 million in costs. In addition to better inpatient care, improved access to ambulatory care may represent an opportunity to reduce costly readmissions among surgical patients.

## Key Points

**Question:**

What proportion of readmissions after major surgery are potentially preventable?

**Findings:**

This cohort study using a weighted sample of 1 937 354 patients found that 18% of readmissions within 90 days of hospital discharge were for a potentially preventable cause. Public insurance, compared with private insurance, was associated with an increased risk of potentially preventable readmission.

**Meaning:**

This study suggests that improved access to ambulatory care in the postoperative period, particularly for high-risk patients, may be associated with a substantial reduction in postoperative readmissions after major surgery.

## Teaser

This cohort study uses data from the 2017 National Readmissions Database to evaluate the degree to which readmissions after major surgery are potentially preventable.

## Introduction

Readmissions after surgical procedures are common, costly, and highly variable across hospital systems.^1,2^ With the Patient Protection and Affordable Care Act and subsequent policy revisions by the Centers for Medicaid & Medicare Services came the Hospital Readmissions Reduction Program, which levied penalties of up to 3% of their base payments against hospital systems for higher-than-expected rates of readmissions.^3^ Subsequent studies suggest that these penalties may have led to a reduction in surgical readmissions and, furthermore, that readmissions after surgical care may have reached a floor beyond which further reductions were unlikely.^4^

Patients with common comorbidities, including congestive heart failure (CHF), chronic obstructive pulmonary disease, hypertension, end-stage kidney disease, and diabetes, have been shown to have higher rates of postsurgical complications, but the underlying mechanisms associated with readmissions after surgery are complex and poorly understood.^5^ As such, the degree to which they can potentially be prevented remains unknown. Ambulatory care sensitive conditions (ACSCs) represent a set of medical conditions, defined in 1993, for which hospitalization is thought to be potentially preventable by timely and effective outpatient care.^6^ This framework was adopted by the Agency for Healthcare Research and Quality (AHRQ) as a hospital quality indicator in 2001.^7^

Using this framework, with the addition of several surgically relevant diagnoses, we hypothesized that a substantial proportion of readmissions after surgery would be potentially preventable and, moreover, that the rate of potentially preventable readmissions (PPRs) would vary according to proxies for timely access to ambulatory care, such as health insurance type.

## Methods

### Data Source and Study Design

This retrospective cohort study used data from the Healthcare Cost and Utilization Project (HCUP) National Readmissions Database (NRD). The NRD was developed by the AHRQ and represents the largest readmissions database in the United States. It is inclusive of all payers and constitutes a nationally representative sample of hospital admissions from 28 geographically dispersed states and accounts for approximately 60% of the US population and 58% of all hospitalizations. The database is assembled annually, and each yearly file is composed of inpatient information from more than 15 million admissions, representing more than 35 million admissions when weighted. The NRD uses patient linkage numbers, which allows for the tracking of patients across hospitals within a state. Out-of-state admissions to non-NRD participating states and out-of-state readmissions are not captured. This cohort study was deemed exempt from approval and informed consent by the institutional review board at the University of Michigan because it is a secondary analysis of publicly available deidentified data. This report follows the Strengthening the Reporting of Observational Studies in Epidemiology (STROBE) reporting guideline.^8^

### Study Population

We selected hospitalizations included in the 2017 file with a primary procedure code for coronary artery bypass grafting, open abdominal aortic aneurysm repair, lower extremity peripheral arterial bypass, laparoscopic colon resection, open colon resection, video-assisted thoracoscopic pulmonary lobectomy, open pulmonary lobectomy, total hip arthroplasty, or total knee arthroplasty. The specific *International Statistical Classification of Diseases and Related Health Problems, Tenth Revision, Procedure Coding System* codes included are reported in eTable 1 in the Supplement.

### Variables

Our primary outcome was readmission within 90 days after hospital discharge after any of the aforementioned procedures that we considered potentially preventable. The definition of potentially preventable was purposely conservative and included readmissions with a primary diagnosis code for any of the ACSCs as defined by the AHRQ, with the addition of 3 specific categories: superficial surgical site infection, acute kidney injury, and aspiration pneumonia or pneumonitis.^7,9,10^ These additional conditions represent surgery-specific additions as well as previously established examples of potentially preventable diagnoses.^10^ A summary of the PPR reasons and their frequencies by procedure are presented in eTable 2 in the Supplement. Overlapping admissions were removed from the analysis. Patients with multiple surgical admissions were uncommon, but when this occurred, only the first index admission for the calendar year was included. Time to event was calculated as the number of days between the index admission and the first readmission. The demographic characteristics of the patients included in our analysis were as follows: age, sex, insurance, surgical procedure, elective or emergency procedure, and the comorbidities included in the Charlson Comorbidity Index.^11^

A hospital’s urban or rural setting and teaching status are coded by HCUP into the following groups for a single categorical variable: nonmetropolitan, metropolitan nonteaching, and metropolitan teaching. Specific cutoffs for each of the hospital types are available through HCUP.^12^ Data on the median household income for the patient’s zip code are obtained from Claritas. When the hospital for the index admission differed from the hospital for the readmission, characteristics for the index admission hospital were used in the models.

Hospital cost estimates were generated using the charge data available for each admission as well as the hospital-level cost to charge ratios provided by HCUP. There was a minimal amount of missing data, with less than 0.1% of primary payer data missing and 1.4% of median household income for zip code data missing. All other variables were complete, and no imputation was undertaken.

### Statistical Analysis

Statistical analysis was performed from January 14 to November 30, 2020. All statistics reported represent values from the weighted sample unless otherwise specifically stated. Overall rates of PPRs as well as individual diagnosis categories are presented as percentages. Univariable analysis was completed with the Wald test or χ^2^ tests on the weighted sample. Multivariable logistic regression models were built with clustered variance estimation owing to patients clustering within hospitals. Given our goal of identifying the factors associated with PPR from a population of patients who otherwise would not be readmitted, we excluded patients with non-PPR readmissions from all multivariable models. When alternative definitions of PPRs were investigated in sensitivity analyses, we similarly excluded non-PPR readmissions. All covariate values were based on the data available from the index admission. To account for differences in Medicare eligibility by age, the association of primary payer stratified by age younger than 65 years or 65 years or older was estimated within a single model that included an interaction term between the covariate for primary payer and a categorial variable for age 65 years or older.

Because the NRD is designed by HCUP to generate national estimates with a complex survey method, all analyses were weighted using an HCUP-provided hospitalization-level weight variable that takes into account the NRD’s sampling design as recommended by the AHRQ.^13^ All hypothesis tests were 2-sided with a 5% type I error rate. All analyses were performed using Stata, version 16.1/MP (StataCorp LLC) with Charlson comorbidities assigned using the charlson package.^14^

To assess the degree to which our results were dependent on our definition of PPR, we performed 2 sensitivity analyses with alternative definitions. We built a multivariable logistic regression model with the same covariates as the primary model already described but with the outcome including ACSCs only as initially defined by the AHRQ in 2001.^7,9^ The second analysis involved building the model with the same covariates as the other 2 models, but with the outcome of PPR defined as any readmission with a length of stay of 2 or fewer days. This definition is based on the notion that very short rehospitalizations may be avoidable with better access to high-quality ambulatory care.

## Results

A total of 1 055 152 unweighted patients underwent surgical procedures. The weighted sample corresponds to an estimation sample of 1 937 354 patients (1 048 046 women [54.1%]; mean age, 66.1 years [95% CI, 66.0-66.3 years]). Of these, 164 755 (8.5%) experienced readmission within 90 days of their index hospitalization. Potentially preventable readmissions accounted for 29 321 (17.8%) of all 90-day readmissions, for an overall PPR rate of 1.5%. The following proportions of patients underwent each of the following procedures: 197 562 (10.1%) underwent coronary artery bypass grafting, 11 144 (0.5%) underwent open abdominal aortic aneurysm repair, 45 181 (2.3%) underwent lower extremity peripheral arterial bypass, 94 082 (4.9%) underwent laparoscopic colon resection, 186 066 (9.5%) underwent open colon resection, 25 506 (1.4%) underwent video-assisted thoracoscopic pulmonary lobectomy, 42 251 (2.2%) underwent open pulmonary lobectomy, 549 138 (28.9%) underwent total hip arthroplasty, and 776 202 (40.1%) underwent total knee arthroplasty. The median hospital cost for all 90-day readmissions was $8310.96 (interquartile range, $5055.26-$14 344.51), with an estimated total hospital cost of approximately $2.01 billion. The median hospital cost for all PPRs within 90 days was $7143.16 (interquartile range, $4647.13-$11 637.56), with an estimated total cost of PPRs of approximately $296 million.

The characteristics of the patients included are reported separately for those who did and for those who did not experience a PPR in Table 1. Patients readmitted for a potentially preventable cause were found to be older (mean age, 71.2 years [95% CI, 70.9-71.4 years]) and less likely to be female (14 444 [49.3%]), with most patients included being treated at large metropolitan teaching hospitals. Patients in the lowest quartile of median household income by zip code had the highest rates of PPR (29.4% [n = 8633]), and patients in the highest quartile had the lowest rates (16.2% [n = 4746]). All the comorbidities investigated through univariable analysis were associated with higher rates of PPR. In addition, patients experiencing a PPR were more likely to have Medicare or Medicaid than private insurance. The most common reasons for PPRs were CHF exacerbation (10 154 of 29 321 [34.6%]), pneumonia (3531 of 29 321 [12.0%]), and acute kidney injury (6593 of 29 321 [22.5%]).

**Table 1.** Patient and Procedure Characteristics

Characteristic	Patients, No. (%)		
	No readmission (n = 1 772 599)	90-d Readmission (n = 164 755)	
		Without PPR (n = 135 434)	With PPR (n = 29 321)
Age, mean (95% CI), y	66.0 (65.8-66.1)	66.9 (66.6-67.2)	71.2 (70.9-71.4)
Female	965 537 (54.5)	68 065 (50.2)	14 444 (49.3)
Surgical procedure			
CABG	167 915 (9.5)	21 610 (16.0)	8037 (27.4)
Open abdominal aortic aneurysm repair	9294 (0.5)	1529 (1.1)	321 (1.1)
Lower extremity bypass	34 083 (1.9)	8717 (6.4)	2381 (8.1)
Colon resection			
Laparoscopic	83 172 (4.7)	9668 (7.1)	1243 (4.2)
Open	152 997 (8.7)	28 382 (20.9)	4686 (16.0)
Pulmonary lobectomy			
Video assisted thoracoscopic	22 955 (1.3)	2129 (1.6)	422 (1.4)
Open	36 728 (2.1)	4500 (3.3)	1022 (3.5)
Total hip arthroplasty	512 084 (29.1)	30 009 (22.2)	7045 (24.0)
Total knee arthroplasty	743 104 (42.2)	28 932 (21.4)	4166 (14.2)
Insurance status			
Medicare	1 019 768 (57.6)	87 550 (64.7)	22 456 (76.6)
Medicaid	97 933 (5.5)	11 260 (8.3)	2058 (7.0)
Private insurance	584 752 (33.0)	31 494 (23.3)	3893 (13.3)
Self-pay	16 520 (0.9)	1637 (1.2)	303 (1.0)
No charge	2358 (0.1)	312 (0.2)	42 (0.1)
Other	49 608 (2.8)	3153 (2.3)	560 (1.9)
Hospital			
Metropolitan			
Nonteaching	427 547 (24.1)	30 295 (22.4)	6835 (23.3)
Teaching	1 191 585 (67.2)	95 166 (70.3)	19 999 (68.2)
Nonmetropolitan	153 410 (8.7)	10 016 (7.4)	2490 (8.5)
Hospital size			
Small	384 446 (21.7)	22 055 (16.3)	4779 (16.3)
Medium	483 891 (27.3)	36 519 (27.0)	8206 (28.0)
Large	904 204 (51.0)	76 903 (56.8)	16 339 (55.7)
Median household income quartile of patient zip code, %			
<25	404 679 (22.8)	36 203 (26.7)	8633 (29.4)
25 to <50	493 841 (27.9)	38 447 (28.4)	8542 (29.1)
50 to <75	467 251 (26.3)	33 696 (24.9)	7082 (24.2)
≥75	383 738 (21.7)	25 650 (18.9)	4746 (16.2)
Emergency surgery	376 445 (21.2)	97 804 (72.2)	23 069 (78.7)
Comorbidities			
Acute myocardial infarction	139 407 (7.9)	16 948 (12.5)	5610 (19.1)
Congestive heart failure	141 866 (8.0)	23 339 (17.2)	12 581 (42.9)
Peripheral vascular disease	122 667 (6.9)	18 006 (13.3)	4978 (17.0)
Cerebrovascular disease	46 677 (2.6)	8137 (6.0)	1921 (6.6)
Dementia	43 974 (2.5)	7945 (5.9)	2548 (8.7)
COPD	310 456 (17.5)	31 456 (23.2)	9660 (33.0)
Rheumatoid disease	61 413 (3.5)	5332 (3.9)	1130 (3.9)
Peptic ulcer disease	8974 (0.5)	2583 (1.9)	321 (1.1)
Mild liver disease	30 329 (1.7)	3650 (2.7)	703 (2.4)
Diabetes	272 529 (15.4)	22 310 (16.5)	5500 (18.8)
Diabetes with complications	121 377 (6.9)	17 311 (12.8)	7847 (26.8)
Hemiplegia or paraplegia	6086 (0.3)	1620 (1.2)	200 (0.7)
Chronic kidney disease	163 824 (9.2)	22 830 (16.9)	9889 (33.7)
Cancer	112 255 (6.3)	13 489 (10.0)	2588 (8.8)
Moderate to severe liver disease	2871 (0.2)	899 (0.7)	163 (5.6)
Metastatic cancer	40 365 (2.3)	7587 (5.6)	1111 (3.8)
AIDS	1700 (0.1)	236 (0.2)	65 (0.2)

Abbreviations: CABG, coronary artery bypass grafting; COPD, chronic obstructive pulmonary disease; PPR, potentially preventable readmission.

**Figure 1.**
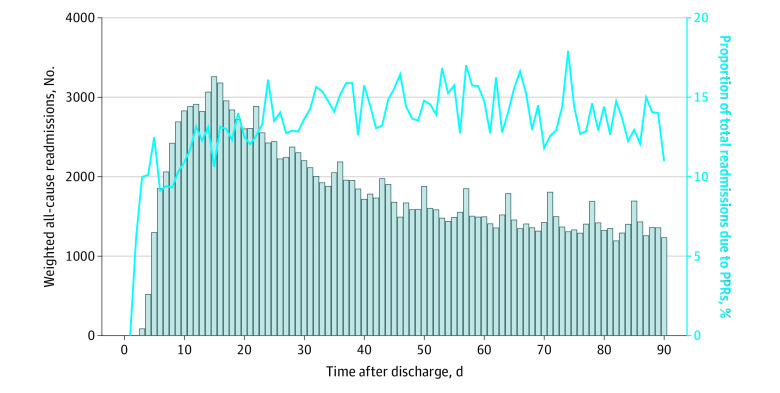
Readmissions Within 90 Days of Hospital Discharge and the Proportion of Potentially Preventable Readmissions (PPRs) The gray bars represent a histogram with each bar representing the aggregate number of readmissions for that number of days after hospital discharge. The solid blue line represents the corresponding proportion of all readmissions for that day due to potentially preventable reasons.

Total readmissions peaked on postdischarge day 15, with 3271 patients readmitted (Figure 1). The proportion of readmissions that were potentially preventable varied considerably with time. The overall readmission rate was 3.6% (n = 67 663) at 30 days and 8.5% (n = 164 755) at 90 days. The proportion of readmissions that were due to potentially preventable reasons was 14.2% at 30 days compared with 17.8% at 90 days.

**Figure 2.**
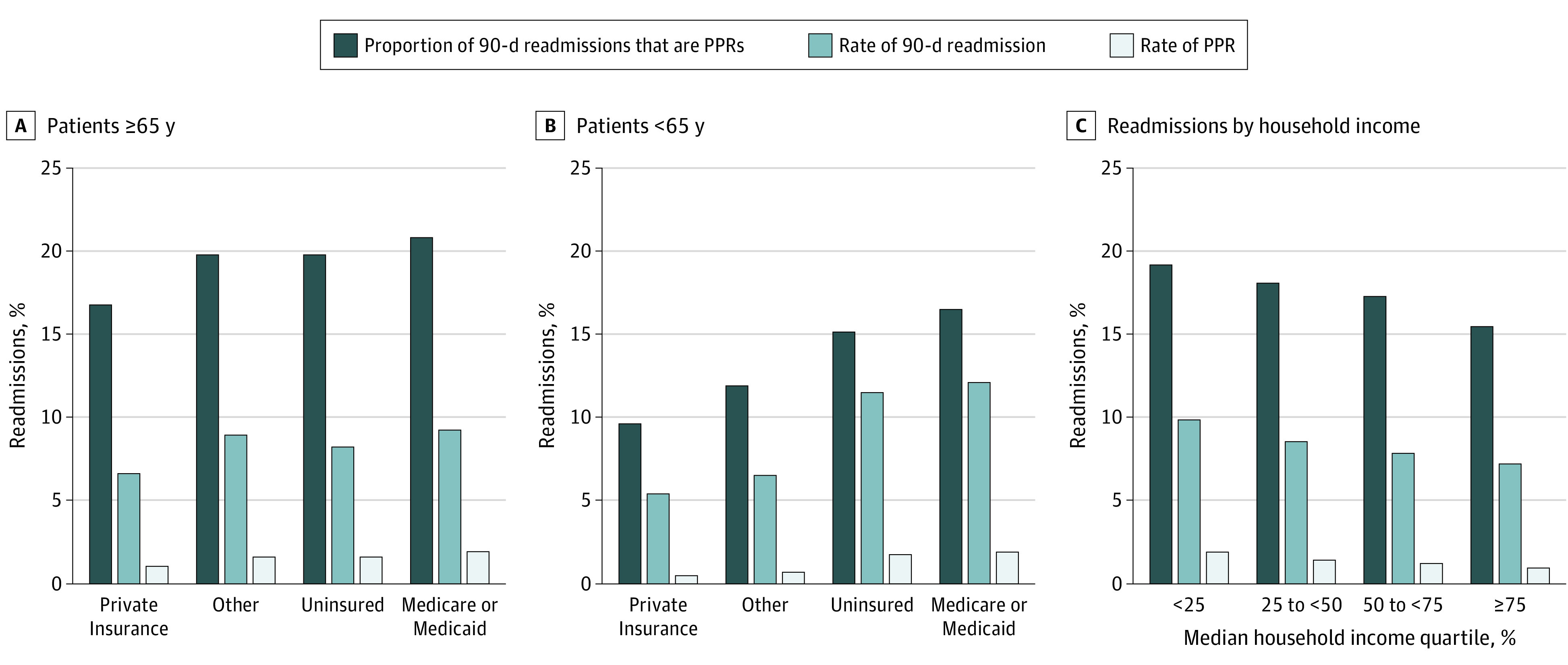
Rates of Potentially Preventable Readmissions (PPRs) and 90-Day Readmissions and the Proportion of Total 90-Day Readmissions Due to Potentially Preventable Causes A, Overall 90-day readmission rate, overall PPR rate, and proportion of 90-day readmissions due to potentially preventable causes for patients aged 65 years or older. B, Overall 90-day readmission rate, overall PPR rate, and proportion of 90-day readmissions due to potentially preventable causes for patients younger than 65 years. C, Overall 90-day readmission rate, overall PPR rate, and proportion of 90-day readmissions due to potentially preventable causes stratified by median household income quartile.

Readmissions and PPRs varied substantially by insurance type (Figure 2A and B). For patients 65 years or older, those with public insurance had the highest rates of both overall 90-day readmissions (9.4% [96 467 of 1 022 602]) and PPRs (2.0% [20 038 of 1 022 602]), while patients with private insurance had the lowest rates of 90-day readmissions (6.8% [6659 of 98 582]) and PPRs (1.1% [1125 of 98 582]). For patients younger than 65 years, those with public insurance also had the highest rates of both 90-day readmissions (12.2% [26 917 of 220 118]) and PPRs (2.0% [4476 of 220 118]), while those with private insurance had the lowest rates of 90-day readmissions (5.4% [28 731 of 522 555]) and PPRs (0.5% [2774 of 522 555]).

A significant association was found between median household income quartile by zip code and rates of 90-day readmissions, PPRs, and the proportion of readmissions due to potentially preventable causes. Patients in the lowest income quartile experienced the highest rates of 90-day readmissions (10.0% [44 836 of 449 515]), decreasing across quartiles to a low of 7.3% (30 396 of 414 134) for those in the top quartile (Figure 2C). A similar association was found for both the overall rate of PPRs (1.9% [8633 of 449 515] for patients in the bottom quartile, decreasing to 1.1% [4746 of 414 134] among those in the top quartile) and the proportion of readmissions due to potentially preventable causes (19.3% [8633 of 44 836] for those in the bottom quartile, decreasing to 15.6% [4746 of 30 396] among those in the top quartile).

Several variables were found to be independently associated with PPR and are reported in Table 2. Each additional decade increase in age was associated with a 20% increase in odds of PPR (adjusted odds ratio [aOR], 1.20; 95% CI, 1.17-1.22), and female sex was associated with a 6% increase in odds of PPR (aOR, 1.06; 95% CI, 1.02-1.10). Furthermore, patients treated at metropolitan teaching hospitals had 8% lower odds of PPR compared with patients in a metropolitan nonteaching hospital (aOR, 0.92; 95% CI, 0.85-0.99). For adult patients younger than 65 years, public insurance was associated with a greater than 2-fold increased odds of PPR compared with those with private insurance (aOR, 2.09; 95% CI, 1.94-2.25). Among patients aged 65 years or older, those with private insurance had 18% lower odds of PPR compared with patients with Medicare as the primary payer (aOR, 0.82; 95% CI, 0.74-0.90). For patients aged 65 years or older, use of Medicare or Medicaid was associated with 22% increased odds of PPR compared with private insurance (aOR, 1.22; 95% CI, 1.11-1.35). Emergency surgery was associated with a more than 8-fold increase in the odds of PPR (aOR, 8.24; 95% CI, 7.63-8.92), and several of the comorbidities investigated were associated with increased odds of PPR, including CHF (aOR, 2.99; 95% CI, 2.85-3.13), dementia (aOR, 1.19; 95% CI, 1.11-1.28), chronic obstructive pulmonary disease (aOR, 1.51; 95% CI, 1.45-1.57), rheumatoid disease (aOR, 1.15; 95% CI, 1.04-1.27), diabetes (aOR, 1.54; 95% CI, 1.46-1.62), chronic kidney disease (aOR, 1.77; 95% CI, 1.68-1.85), liver disease (aOR, 1.31; 95% CI, 1.02-1.69), metastatic cancer (aOR, 1.15; 95% CI, 1.03-1.28), and AIDS (aOR, 1.87; 95% CI, 1.25-2.79).

**Table 2.** Multivariable Logistic Regression Model for Potentially Preventable Readmissions

Characteristic	aOR (95% CI)	*P* value
Age, per additional decade	1.20 (1.17-1.22)	<.001
Female	1.06 (1.02-1.10)	.002
Surgical procedure		
CABG	1 [Reference]	NA
Open abdominal aortic aneurysm repair	1.34 (1.10-1.63)	.003
Lower extremity bypass	1.69 (1.52-1.88)	<.001
Colon resection		
Laparoscopic	0.93 (0.83-1.04)	.19
Open	0.89 (0.82-0.97)	.008
Pulmonary lobectomy		
Video assisted thoracoscopic	1.00 (0.84-1.17)	.95
Open	1.72 (1.52-1.95)	<.001
Total hip arthroplasty	0.51 (0.47-0.55)	<.001
Total knee arthroplasty	0.58 (0.52-0.64)	<.001
Insurance status^a^		
For patients aged <65 y		
Private insurance	1 [Reference]	NA
Medicare or Medicaid	2.09 (1.94-2.25)	<.001
Uninsured	1.12 (0.92-1.38)	.27
Other	1.19 (1.00-1.42)	.05
For patients aged ≥65 y		
Private insurance	1 [Reference]	NA
Medicare or Medicaid	1.22 (1.11-1.35)	<.001
Uninsured	0.82 (0.54-1.24)	.34
Other	0.98 (0.79-1.21)	.84
Hospital		
Metropolitan		
Nonteaching	1 [Reference]	NA
Teaching	0.92 (0.85-0.99)	.03
Nonmetropolitan	1.05 (0.95-1.18)	.34
Hospital size		
Small	1 [Reference]	NA
Medium	0.95 (0.86-1.05)	.29
Large	0.89 (0.82-0.98)	.01
Median household income quartile of patient zip code, %		
<25	1 [Reference]	NA
25 to <50	0.95 (0.90-1.00)	.06
50 to <75	0.93 (0.87-0.98)	.01
≥75	0.83 (0.77-0.89)	<.001
Emergency surgery	8.24 (7.63-8.92)	<.001
Comorbidities		
Acute myocardial infarction	0.60 (0.57-0.65)	<.001
Congestive heart failure	2.99 (2.85-3.13)	<.001
Peripheral vascular disease	0.89 (0.84-0.95)	<.001
Cerebrovascular disease	0.89 (0.83-0.97)	.006
Dementia	1.19 (1.11-1.28)	<.001
COPD	1.51 (1.45-1.57)	<.001
Rheumatoid disease	1.15 (1.04-1.27)	.004
Peptic ulcer disease	0.96 (0.81-1.14)	.65
Mild liver disease	0.96 (0.85-1.08)	.48
Diabetes	1.54 (1.46-1.62)	<.001
Diabetes with complications	1.80 (1.70-1.90)	<.001
Hemiplegia or paraplegia	0.79 (0.62-1.01)	.06
Chronic kidney disease	1.77 (1.68-1.85)	<.001
Cancer	1.04 (0.96-1.12)	.39
Moderate to severe liver disease	1.31 (1.02-1.69)	.03
Metastatic cancer	1.15 (1.03-1.28)	.009
AIDS	1.87 (1.25-2.79)	.002

Abbreviations: aOR, adjusted odds ratio; CABG, coronary artery bypass grafting; COPD, chronic obstructive pulmonary disease; NA, not applicable.

^a^ Association of primary payer stratified by age younger than 65 years or 65 years or older was estimated with a single model including an interaction term between the covariate for primary payer and a categorial variable for age 65 years or older.

**Figure 3.**
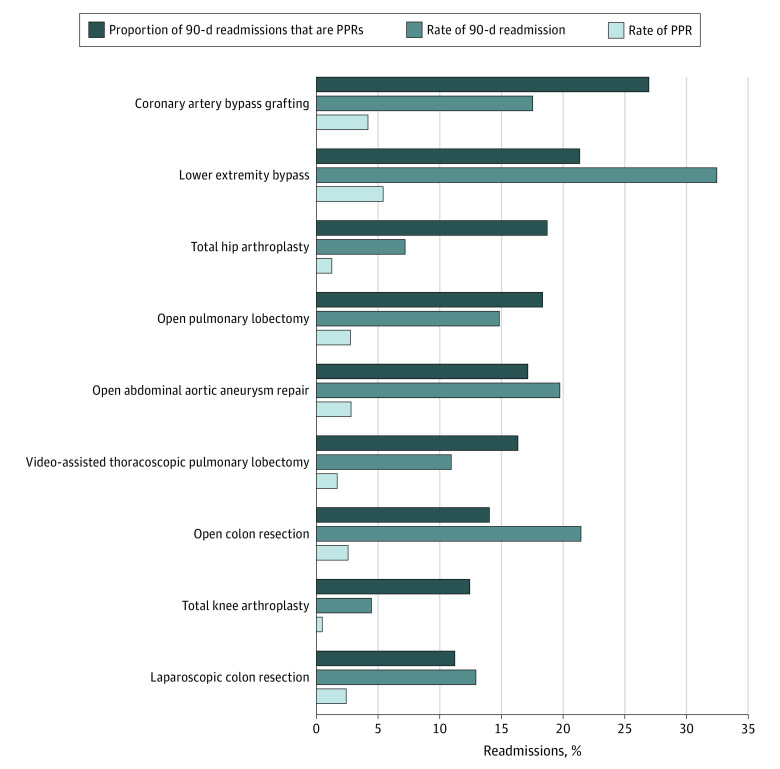
Procedure-Specific Rates of Potentially Preventable Readmissions (PPRs) and 90-Day Readmissions and the Proportion of Total 90-Day Readmissions Due to Potentially Preventable Causes Procedures are ordered from top to bottom, with decreasing proportion of readmissions due to potentially preventable causes.

Overall PPR rates varied significantly across the surgical procedures investigated, with the lowest PPR rate found for total knee arthroplasty (0.5% [4166 of 776 202]) and the highest rate for lower extremity peripheral arterial bypass (5.6% [2381 of 45 181]). Rates of PPRs are reported in Figure 3. Data on the frequencies for each PPR reason by procedure type are available in eTable 3 in the Supplement.

To investigate the degree to which our analysis was dependent on our outcome definition, we completed 2 sensitivity analyses. First, we altered our outcome definition to include only ACSCs. Overall, similar estimates were found for most covariates (eTable 4 in the Supplement). The estimate for the association of open abdominal aortic aneurysm repair compared with coronary artery bypass grafting became nonsignificant, while the association for both laparoscopic and open colon resection increased in magnitude. A second sensitivity analysis was completed with the outcome of PPR defined as a length of stay of 2 or fewer days. Age became nonsignificant, and female sex became protective. Laparoscopic and open colon resections as well as total knee arthroplasties were associated with increased odds of PPR rather than the decreased odds found in the primary analysis. The association of comorbidities was different as well, with significant associations found for several comorbidities that are not associated with ACSCs. The results of this model are shown in eTable 5 in the Supplement. The association between PPRs and both health insurance and income quartile remained similar across the main analysis and both sensitivity analyses.

## Discussion

In this study of readmissions from the NRD, we found that nearly 1 in 5 readmissions after these selected major surgical procedures were potentially preventable, with an estimated cost to hospitals of nearly $300 million annually. We also found that patients who were readmitted for potentially preventable reasons had worse underlying health, as evidenced by our findings that these patients were older and more likely to have a variety of comorbidities, most notably those that are directly associated with ACSC such as CHF, chronic obstructive pulmonary disease, chronic kidney disease, or diabetes. Finally, the likelihood of PPRs was closely associated with both income and health insurance coverage.

Medicare or Medicaid as the primary payer was found to be associated with increased rates of PPR compared with private insurance. Although the direct cause of the association between primary payer and risk of PPR cannot be ascertained using this data source, previous studies have shown that government-provided insurance through Medicare or Medicaid and lower socioeconomic status are markers of clinical risk but also of poorer access to primary care relative to those with private insurance.^6,15,16,17,18,19,20^ Future work must separate the association of increased clinical risk from the direct association of access to care and medical management with various insurance types. Together, our findings suggest that PPRs after surgery represent a significant burden to the US health care system and that interventions to improve PPR rates may be targeted to patients with significant underlying comorbidities as well as those undergoing high-risk operations.

This study expands on prior work in several ways. Prior studies investigating postoperative readmissions have shown that approximately 1 in 4 Medicare beneficiaries undergoing surgery is readmitted within 90 days of hospital discharge, but the reasons for readmission have not been well elucidated.^1^ We used the all-payer NRD, which captures the full scope of patients undergoing surgical procedures in the United States and allows for a national estimate of health system burden from PPRs as well as robust cost estimates. Our results suggest that potentially preventable causes account for at least 17% of readmissions within the 90-day postoperative period investigated and, furthermore, that the proportion of readmissions for these reasons did not substantially vary during the course of the postoperative period. This finding suggests that undergoing surgery increases the risk of hospital admission due to these underlying medical problems rather than only surgery-specific reasons. Prior investigations into ACSCs have been limited to medical patients, making this the first assessment of ACSC admissions in the postoperative setting, to our knowledge.^21,22,23,24,25^ Studies investigating the causes of readmission and attempts to predict those at highest risk have not specifically addressed causes that may be potentially preventable but have investigated all causes of readmission or those for surgical complications, potentially missing an opportunity to target readmission interventions to those that might be most likely to be prevented.^26,27,28^

Criticisms against using readmissions as a quality measure in a postsurgical population compared with the medical population, in which they are much more widely accepted, have been centered around the fact that medical readmissions are more commonly associated with suboptimal transition of care from the hospital to the outpatient setting.^29^ This finding contrasts with surgical readmissions, for which risks may be inherent to the operation and its complications and therefore less directly associated with hospital quality.^30^ The difficulty in disentangling the relative responsibility of hospital and patient factors in optimizing the transition from inpatient to outpatient care in the postoperative period, and its applicability to complication-associated readmissions, has traditionally limited enthusiasm for use of readmissions as a quality indicator for surgical patients. Our results, however, suggest that a substantial proportion of readmissions in the postoperative setting may be associated with factors strongly associated with transitions from inpatient care to outpatient care rather than inherent risks associated with the procedure or its complications. In this way, postoperative readmissions are not merely a measure of hospital and inpatient care quality but also represent a measure of access to and quality of ambulatory care after hospital discharge. Furthermore, our finding of significant variation in PPR rates across a variety of socioeconomic factors and based on primary payer status, as proxies for access to care, suggests that improved access to ambulatory care may result in decreased rates of readmissions for these potentially preventable causes and a significant savings in cost and burden to the health care system as a whole.

### Limitations

Our results should be interpreted within the context of several limitations. The NRD contains little clinical information about the operative procedures, the hospitalizations, or the readmissions. It remains possible that significant unmeasured bias is present in our estimates for the covariates investigated, particularly for comorbidities for which significant clinical heterogeneity is present, such as CHF, chronic kidney disease, and diabetes. In addition, complication rates for all surgical procedures are associated with patients’ underlying health status. In this way, the actual association with risk of PPR may not be due to patients’ underlying comorbidities or poorer access to care but instead may be simply caused by increased rates of postoperative complications. Our results, however, suggest that there is a particular association of comorbidities with a direct association with ACSCs, such as CHF and chronic kidney disease, which supports the hypothesis that the associations with readmissions due to surgical complications and readmissions for medical diagnoses, such as ACSCs, may be separable. The disentanglement of underlying comorbidities, access to care, and postoperative complications will require further research with more clinical granularity.

## Conclusions

Our results demonstrate that, of the 8.5% of patients who were readmitted within 90 days after several common surgical procedures, 17.8% of such readmission were for reasons that may be preventable with improved access to outpatient services. Furthermore, having several comorbidities, a lower income, and a public primary payer were associated with readmission for potentially preventable reasons, suggesting that targeted interventions to this high-risk group of patients may be a potential pathway to decreasing postoperative readmissions and may result in substantial cost savings to the US health care system.

## Notes

Supplement. **eTable 1.** *ICD-10-PCS* Codes for Definition of Surgical Admissions **eTable 2.** Top 15 Diagnosis Codes for 90-Day Readmissions **eTable 3.** 90-Day Readmissions **eTable 4.** Sensitivity Analysis—Multivariable Model—ACSC Only **eTable 5.** Sensitivity Analysis—Multivariable Model—LOS ≤ 2 Days
