# Supplementary material for: Assessment of Potentially Preventable Hospital Readmissions After Major Surgery and Association With Public vs Private Health Insurance and Comorbidities
Source: JAMA Netw Open. 2021 Apr 13;4(4):e215503. doi: 10.1001/jamanetworkopen.2021.5503 (PMC8044735; doi:10.1001/jamanetworkopen.2021.5503)
Supplement: Supplement. — eTable 1. ICD-10-PCS Codes for Definition of Surgical Admissions eTable 2. Top 15 Diagnosis Codes for 90-Day Readmissions eTable 3. 90-Day Readmissions eTable 4. Sensitivity Analysis—Multivariable Model—ACSC Only eTable 5. Sensitivity Analysis—Multivariable Model—LOS ≤ 2 Days [file jamanetwopen-e215503-s001.pdf]

Supplementary Online Content

Brown CS, Montgomery JR, Neiman PU, et al. Assessment of potentially preventable hospital readmissions after major surgery and association with public vs private health insurance and comorbidities. *JAMA Netw Open.* 2021;4(4):e215503. doi:10.1001/jamanetworkopen.2021.5503

eTable 1. *ICD-10-PCS* Codes for Definition of Surgical Admissions

eTable 2. Top 15 Diagnosis Codes for 90-Day Readmissions

eTable 3. 90-Day Readmissions

eTable 4. Sensitivity Analysis—Multivariable Model—ACSC Only

eTable 5. Sensitivity Analysis—Multivariable Model—LOS ≤ 2 Days

This supplementary material has been provided by the authors to give readers additional information about their work.

© 2021 Brown CS et al. *JAMA Network Open.*

eTable 1. *ICD-10-PCS* Codes for Definition of Surgical Admissions

Coronary Artery Bypass Grafting

0210083	0213083	02100J3	02100ZF	02110J9	021209F	02120KW	02130J8
0210088	0213088	02100J3	02104A3	02110JC	021209W	02120Z3	02130J9
0210089	0213089	02100J8	02104J3	02110JF	02120A3	02120Z8	02130JC
0210093	0213093	02100J9	02104K3	02110JW	02120A8	02120Z9	02130JF
0210098	0213098	02100JC	02104Z3	02110K3	02120A9	02120ZC	02130JW
0210099	0213099	02100JF	021108C	02110K8	02120AC	02120ZF	02130K3
0210493	021008C	02100JW	021108F	02110K9	02120AF	021308C	02130K8
0211083	021008F	02100K3	021108W	02110KC	02120AW	021308F	02130K9
0211088	021008W	02100K3	021109C	02110KF	02120J3	021308W	02130KC
0211089	021009C	02100K8	021109F	02110KW	02120J8	021309C	02130KF
0211093	021009F	02100K9	021109W	02110Z3	02120J9	021309F	02130KW
211098	021009W	02100KC	02110A3	02110Z8	02120JC	021309W	02130Z3
0211099	02100A3	02100KF	02110A8	02110Z9	02120JF	02130A3	02130Z8
0212083	02100A3	02100KW	02110A9	02110ZC	02120JW	02130A8	02130Z9
0212088	02100A8	02100Z3	02110AC	02110ZF	02120K3	02130A9	02130ZC
0212089	02100A9	02100Z3	02110AF	021208C	02120K8	02130AC	02130ZF
0212093	02100AC	02100Z8	02110AW	021208F	02120K9	02130AF	
0212098	02100AF	02100Z9	02110J3	021208W	02120KC	02130AW	
0212099	02100AW	02100ZC	0110J8	021209C	02120KF	02130J3	

Open Abdominal Aortic Aneurysm Repair

04B00ZZ	041009Q	04100J8	04100KD	04100ZJ	041C0ZK	041E09K	041F0AK
04R007Z	041009R	04100J9	04100KF	04100ZK	041D09H	041E0AH	041F0JH
04R00JZ	04100A6	04100JB	04100KG	04100ZQ	041D09J	041E0AJ	041F0JJ
04R00KZ	04100A7	04100JC	04100KH	04100ZR	041D09K	041E0AK	041F0JK
04500ZZ	04100A8	04100JD	04100KJ	041C09H	041D0AH	041E0JH	041F0KH
04B00ZZ	04100A9	04100JF	04100KK	041C09J	041D0AJ	041E0JJ	041F0KJ
0410096	04100AB	04100JG	04100KQ	041C09K	041D0AK	041E0JK	041F0KK
0410097	04100AC	04100JH	04100KR	041C0AH	041D0JH	041E0KH	041F0ZH
0410098	04100AD	04100JJ	04100Z6	041C0AJ	041D0JJ	041E0KJ	041F0ZJ
0410099	04100AF	04100JK	04100Z7	041C0AK	041D0JK	041E0KK	041F0ZK
041009B	04100AG	04100JQ	04100Z8	041C0JH	041D0KH	041E0ZH	041H09H
041009C	04100AH	04100JR	04100Z9	041C0JJ	041D0KJ	041E0ZJ	041H09J
041009D	04100AJ	04100K6	04100ZB	041C0JK	041D0KK	041E0ZK	041H09K
041009F	04100AK	04100K7	04100ZC	041C0KH	041D0ZH	041F09H	041H0AH
041009G	04100AQ	04100K8	04100ZD	041C0KJ	041D0ZJ	041F09J	041H0AJ
041009H	04100AR	04100K9	04100ZF	041C0KK	041D0ZK	041F09K	041H0AK
041009J	04100J6	04100KB	04100ZG	041C0ZH	041E09H	041F0AH	041H0JH
041009K	04100J7	04100KC	04100ZH	041C0ZJ	041E09J	041F0AJ	041H0JJ

© 2021 Brown CS et al. *JAMA Network Open.*

041H0JK	041H0KK	041H0ZK	041J09K	041J0AK	041J0JK	041J0KK	041J0ZJ
041H0KH	041H0ZH	041J09H	041J0AH	041J0JH	041J0KH	041J0ZH	041J0ZK
041H0KJ	041H0ZJ	041J09J	041J0AJ	041J0JJ	041J0KJ		

Lower Extremity Bypass

041K09H	041K0JK	041K0ZM	041L0AP	041L0KS	041M0JL	041N0AL
041K09J	041K0JL	041K0ZN	041L0AQ	041L0ZH	041M0JM	041N0AM
041K09K	041K0JM	041K0ZP	041L0AS	041L0ZJ	041M0JP	041N0AP
041K09L	041K0JN	041K0ZQ	041L0JH	041L0ZK	041M0JQ	041N0AQ
041K09M	041K0JP	041K0ZS	041L0JJ	041L0ZL	041M0JS	041N0AS
041K09N	041K0JQ	041L09H	041L0JK	041L0ZM	041M0KL	041N0JL
041K09P	041K0JS	041L09J	041L0JL	041L0ZN	041M0KM	041N0JM
041K09Q	041K0KH	041L09K	041L0JM	041L0ZP	041M0KP	041N0JP
041K09S	041K0KJ	041L09L	041L0JN	041L0ZQ	041M0KQ	041N0JQ
041K0AH	041K0KK	041L09M	041L0JP	041L0ZS	041M0KS	041N0JS
041K0AJ	041K0KL	041L09N	041L0JQ	041M09L	041M0ZL	041N0KL
041K0AK	041K0KM	041L09P	041L0JS	041M09M	041M0ZM	041N0KM
041K0AL	041K0KN	041L09Q	041L0KH	041M09P	041M0ZP	041N0KP
041K0AM	041K0KP	041L09S	041L0KJ	041M09Q	041M0ZQ	041N0KQ
041K0AN	041K0KQ	041L0AH	041L0KK	041M09S	041M0ZS	041N0KS
041K0AP	041K0KS	041L0AJ	041L0KL	041M0AL	041N09L	041N0ZL
041K0AQ	041K0ZH	041L0AK	041L0KM	041M0AM	041N09M	041N0ZM
041K0AS	041K0ZJ	041L0AL	041L0KN	041M0AP	041N09P	041N0ZP
041K0JH	041K0ZK	041L0AM	041L0KP	041M0AQ	041N09Q	041N0ZQ
041K0JJ	041K0ZL	041L0AN	041L0KQ	041M0AS	041N09S	041N0ZS

Laparoscopic Colon Resection

0DTH4ZZ	0DTL4ZZ	0DTN4ZZ	0DBF4ZZ	0DBH4ZZ	0DBL4ZZ	0DBN4ZZ	0DTM4ZZ
0DTF4ZZ	0DTG4ZZ	0DBE4ZZ	0DBG4ZZ	0DBK4ZZ	0DBM4ZZ	0DTK4ZZ	0DTE4ZZ

Open Colon Resection

0DBE0ZZ	0DTK0ZZ	0DTN0ZZ	0DBG0ZZ	0DBL0ZZ	0DTK0ZZ
0DTH0ZZ	0DTL0ZZ	0DBE0ZZ	0DBH0ZZ	0DBM0ZZ	0DTM0ZZ
0DTF0ZZ	0DTG0ZZ	0DBF0ZZ	0DBK0ZZ	0DBN0ZZ	0DTE0ZZ

Thoracoscopic Pulmonary Lobectomy

0BBC4ZZ
0BBD4ZZ

© 2021 Brown CS et al. *JAMA Network Open.*

0BBF4ZZ
0BBG4ZZ
0BBJ4ZZ

Open Pulmonary Lobectomy

0B5C0ZZ	0B5D7ZZ	0B5G3ZZ	0BBC0ZZ	0BBD7ZZ	0BBG3ZZ	0BTC4ZZ	0BTC0ZZ
0B5C3ZZ	0B5F0ZZ	0B5G7ZZ	0BBC3ZZ	0BBF0ZZ	0BBG7ZZ	0BTD4ZZ	0BTD0ZZ
0B5C7ZZ	0B5F3ZZ	0B5J0ZZ	0BBC7ZZ	0BBF3ZZ	0BBJ0ZZ	0BTF4ZZ	0BTF0ZZ
0B5D0ZZ	0B5F7ZZ	0B5J3ZZ	0BBD0ZZ	0BBF7ZZ	0BBJ3ZZ	0BTG4ZZ	0BTG0ZZ
0B5D3ZZ	0B5G0ZZ	0B5J7ZZ	0BBD3ZZ	0BBG0ZZ	0BBJ7ZZ	0BTJ4ZZ	0BTJ0ZZ

Total Hip Arthoplasty

0SR9019	0SR904Z	0SRB02Z	0SRB0KZ	0SRA0J9	0SRE03A	0SRR03A	0SRS03A
0SR901A	0SR907Z	0SRB039	0SRA009	0SRA0JA	0SRE03Z	0SRR03Z	0SRS03Z
0SR901Z	0SR90J9	0SRB03A	0SRA00A	0SRA0JZ	0SRE07Z	0SRR07Z	0SRS07Z
0SR9029	0SR90JA	0SRB03Z	0SRA00Z	0SRA0KZ	0SRE0J9	0SRR0J9	0SRS0J9
0SR902A	0SR90JZ	0SRB049	0SRA019	0SRE009	0SRE0JA	0SRR0JA	0SRS0JA
0SR902Z	0SR90KZ	0SRB04A	0SRA01A	0SRE00A	0SRE0JZ	0SRR0JZ	0SRS0JZ
0SR9039	0SRB019	0SRB04Z	0SRA01Z	0SRE00Z	0SRE0KZ	0SRR0KZ	0SRS0KZ
0SR903A	0SRB01A	0SRB07Z	0SRA039	0SRE019	0SRR019	0SRS019	
0SR903Z	0SRB01Z	0SRB0J9	0SRA03A	0SRE01A	0SRR01A	0SRS01A	
0SR9049	0SRB029	0SRB0JA	0SRA03Z	0SRE01Z	0SRR01Z	0SRS01Z	
0SR904A	0SRB02A	0SRB0JZ	0SRA07Z	0SRE039	0SRR039	0SRS039	

Total Knee Arthoplasty

0SRC07Z	0SRD0LZ	0SRV0KZ
0SRC0J9	0SRT07Z	0SRW07Z
0SRC0JA	0SRT0J9	0SRW0J9
0SRC0JZ	0SRT0JA	0SRW0JA
0SRC0KZ	0SRT0JZ	0SRW0JZ
0SRC0L9	0SRT0KZ	0SRW0KZ
0SRC0LA	0SRU07Z	
0SRC0LZ	0SRU0J9	
0SRD07Z	0SRU0JA	
0SRD0J9	0SRU0JZ	
0SRD0JA	0SRU0KZ	
0SRD0JZ	0SRV07Z	
0SRD0KZ	0SRV0J9	
0SRD0L9	0SRV0JA	
0SRD0LA	0SRV0JZ	

© 2021 Brown CS et al. *JAMA Network Open.*

**eTable 2.** Top 15 Diagnosis Codes for 90-Day Readmissions	
**Primary Diagnosis for Readmission**	**Readmissions (n=164,755), n(%)**
Infection following a procedure, initial encounter	7097 (6.6)
Sepsis, unspecified organism	6420 (6.0)
**Acute Kidney Failure, unspecified**	3224 (3.0)
**Hypertensive Heart Disease with Heart Failure**	2437 (2.3)
**Hypertensive heart and chronic kidney disease with heart failure and stage 1 through stage 4 chronic kidney disease, or unspecified chronic kidney disease**	2099 (2.0)
Other pulmonary embolism without acute cor pulmonale	2032 (1.9)
Encounter for attention to ileostomy	1961 (1.8)
**Pneumonia, unspecified organism**	1572 (1.5)
**Urinary tract infection, site not specified**	1309 (1.2)
Disruption of external operation (surgical) wound, not elsewhere classified, initial encounter	1292 (1.2)
Non-ST elevation (NSTEMI) myocardial infarction	1204 (1.1)
Pleural effusion, not elsewhere classified	1110 (1.0)
Paroxysmal atrial fibrillation	939 (0.9)
Unspecified intestinal obstruction	899 (0.8)
Gastrointestinal hemorrhage, unspecified	894 (0.8)
*Bolded entries represent codes defined as Ambulatory Care Sensitive Conditions (ACSCs)	

© 2021 Brown CS et al. *JAMA Network Open.*

eTable 3. 90-Day Readmissions

	90 Day Readmissions									
**Diagnosis**	**All Surgery**	**CABG**	**AAA**	**LE Bypass**	**Lap Colon**	**Open Colon**	**VATS Lobectomy**	**Open Lobectomy**	**THA**	**TKA**
Any Diagnosis	8.5%	15.0%	16.6%	24.6%	11.6%	17.8%	10.0%	13.1%	6.7%	4.3%
All Potentially Preventable Reasons	1.5%	4.1%	2.9%	5.3%	1.3%	2.5%	1.7%	2.4%	1.3%	0.5%
Short Term Diabetes Complications	0%	0%	0%	0%	0%	0%	0%	0%	0%	0%
Long Term Diabetes Complications	0.1%	0.2%	0.2%	2.6%	0%	0.1%	0%	0%	0%	0%
Chronic Obstructive Pulmonary Disease	0.2%	0.3%	0.5%	0.4%	0.1%	0.2%	0.6%	0.7%	0.2%	0.1%
Hypertension	0%	0.1%	0.1%	0.1%	0%	0%	0%	0.1%	0%	0%
Hypertension-Related Chronic Kidney Disease	0%	0%	0%	0%	0%	0%	0%	0%	0%	0%
Congestive Heart Failure	0.5%	2.6%	0.8%	1.4%	0.2%	0.4%	0.3%	0.4%	0.4%	0.1%
Community Acquired Pneumonia	0.2%	0.4%	0.2%	0.3%	0.1%	0.2%	0.5%	0.7%	0.2%	0.1%
Sickle Cell Disease	0%	0%	0%	0%	0%	0%	0%	0%	0%	0%
Urinary Tract Infection	0.2%	0.2%	0.3%	0.2%	0.2%	0.4%	0.1%	0.2%	0.2%	0.1%
Uncontrolled Diabetes	0%	0.1%	0.1%	0.1%	0%	0.1%	0%	0%	0%	0%
Failure to Thrive	0%	0%	0%	0%	0%	0%	0%	0%	0%	0%
Dental Conditions	0%	0%	0%	0%	0%	0%	0%	0%	0%	0%
Vaccine Preventable Diseases	0%	0%	0%	0%	0%	0%	0%	0%	0%	0%
Nutritional Deficiencies	0%	0%	0.1%	0%	0%	0%	0%	0%	0%	0%
Convulsions	0%	0%	0%	0%	0%	0%	0%	0%	0%	0%

© 2021 Brown CS et al. *JAMA Network Open.*

Dehydration	0%	0%	0%	0%	0%	0%	0%	0%	0%	0%
Hypoglycemia	0%	0%	0%	0%	0%	0%	0%	0%	0%	0%
Pelvic Inflammatory Disease	0%	0%	0%	0%	0%	0%	0%	0%	0%	0%
Ear-Nose-Throat Infections	0%	0%	0%	0%	0%	0%	0%	0%	0%	0%
Angina	0%	0%	0%	0%	0%	0%	0%	0%	0%	0%
Grand Mal Seizures	0%	0%	0%	0%	0%	0%	0%	0%	0%	0%
Superficial Surgical Site Infection	0%	0%	0.1%	0%	0%	0%	0%	0%	0%	0%
Acute Kidney Injury/Acute Renal Failure	0.3%	0.5%	0.7%	0.6%	0.6%	1.2%	0.1%	0.3%	0.2%	0.1%
Aspiration Pneumonitis	0.1%	0.1%	0.1%	0.1%	0%	0.1%	0.1%	0.1%	0.1%	0%

© 2021 Brown CS et al. *JAMA Network Open.*

**eTable 4.** Sensitivity Analysis—Multivariable Model—ACSC Only			
**Characteristic**	**aOR**	**95% CI**	***P* value**
**Age, per additional decade**	1.19	1.17-1.22	<0.001
**Female**	1.14	1.09-1.19	<0.001
**Surgical Procedure**			
Coronary Artery Bypass Grafting	REF	REF	
Open Abdominal Aortic Aneurysm Repair	1.05	0.83-1.31	0.697
Lower Extremity Bypass	1.66	1.49-1.86	<0.001
Laparoscopic Colon Resection	0.53	0.47-0.61	<0.001
Open Colon Resection	0.49	0.45-0.55	<0.001
Video Assisted Thoracoscopic Pulmonary Lobectomy	1.03	0.87-1.22	0.745
Open Pulmonary Lobectomy	1.63	1.41-1.87	<0.001
Total Hip Arthroplasty	0.46	0.42-0.50	<0.001
Total Knee Arthroplasty	0.48	0.43-0.53	<0.001
**Insurance Status**			
For age <65 years			
Private Insurance	REF	REF	
Medicare/Medicaid	2.21	2.03-2.42	<0.001
Uninsured	1.17	0.92-1.48	0.197
Other	1.28	1.04-1.57	0.017
For age ≥ 65 years			
Private Insurance	REF	REF	
Medicare/Medicaid	1.20	1.07-1.34	0.001
Uninsured	0.68	0.40-1.13	0.135
Other	0.83	0.65-1.06	0.129
**Hospital Metropolitan-Teaching Status**			
Metropolitan Non-teaching	REF	REF	
Metropolitan Teaching	0.89	0.82-0.96	0.003
Non-metropolitan	1.09	0.97-1.23	0.149
**Hospital Size**			
Small	REF	REF	
Medium	0.95	0.85-1.05	0.288
Large	0.88	0.80-0.97	0.007
**Median Household Income Quartile of Patient Zip Code**			
<25%	REF	REF	
25-50%	0.95	0.89-1.00	0.061
50-75%	0.92	0.86-0.98	0.021
>75%	0.83	0.77-0.90	<0.001
**Emergent Surgery**	7.81	7.18-8.48	<0.001
**Comorbidities**			

© 2021 Brown CS et al. *JAMA Network Open.*

Acute Myocardial Infarction	0.59	0.55-0.64	<0.001
Congestive Heart Failure	3.68	3.49-3.88	<0.001
Peripheral Vascular Disease	0.94	0.88-1.00	0.039
Cerebrovascular Disease	0.84	0.77-0.91	<0.001
Dementia	1.09	1.00-1.19	0.045
Chronic Obstructive Pulmonary Disease	1.62	1.45-1.70	<0.001
Rheumatoid Disease	1.22	1.10-1.36	<0.001
Peptic Ulcer Disease	0.91	0.74-1.12	0.356
Mild Liver Disease	0.97	0.85-1.12	0.693
Diabetes	1.49	1.41-1.58	<0.001
Diabetes with Complications	1.88	1.77-2.00	<0.001
Hemiplegia or Paraplegia	0.94	0.72-1.23	0.654
Chronic Kidney Disease	1.51	1.43-1.60	<0.001
Cancer	1.04	0.94-1.14	0.484
Moderate to Severe Liver Disease	0.91	0.67-1.22	0.508
Metastatic Cancer	1.06	0.93-1.22	0.394
Acquired Immunodeficiency Syndrome	2.10	1.34-3.28	0.001

*Effect of primary payer stratified by age < or ≥ 65 years was estimated with a single model including an interaction term between the covariate for primary payer and a categorial variable for age ≥ 65 years. Abbreviations: aOR, adjusted odds ratio; CI, confidence interval; ACSC, Ambulatory Care Sensitive Condition

© 2021 Brown CS et al. *JAMA Network Open.*

**eTable 5.** Sensitivity Analysis—Multivariable Model—LOS ≤ 2 Days			
**Characteristic**	**aOR**	**95% CI**	***P* value**
**Age, per additional decade**	1.00	1.00-1.00	0.214
**Female**	0.91	0.89-0.93	<0.001
**Surgical Procedure**			
Coronary Artery Bypass Grafting	REF	REF	
Open Abdominal Aortic Aneurysm Repair	1.25	1.10-1.43	0.001
Lower Extremity Bypass	1.78	1.65-1.93	<0.001
Laparoscopic Colon Resection	1.58	1.46-1.72	<0.001
Open Colon Resection	1.14	1.06-1.23	<0.001
Video Assisted Thoracoscopic Pulmonary Lobectomy	1.00	0.89-1.12	0.961
Open Pulmonary Lobectomy	1.82	1.66-1.99	<0.001
Total Hip Arthroplasty	0.80	0.75-0.85	<0.001
Total Knee Arthroplasty	1.35	1.23-1.47	<0.001
**Insurance Status**			
For age <65 years			
Private Insurance	REF	REF	
Medicare/Medicaid	1.44	1.38-1.50	<0.001
Uninsured	0.75	0.67-0.83	<0.001
Other	0.93	0.85-1.02	0.105
For age ≥ 65 years			
Private Insurance	REF	REF	
Medicare/Medicaid	1.09	1.02-1.15	0.007
Uninsured	0.65	0.47-0.90	0.009
Other	0.91	0.80-1.03	0.144
**Hospital Metropolitan-Teaching Status**			
Metropolitan Non-teaching	REF	REF	
Metropolitan Teaching	1.04	0.97-1.11	0.282
Non-metropolitan	0.94	0.85-1.04	0.231
**Hospital Size**			
Small	REF	REF	
Medium	0.97	0.88-1.08	0.611
Large	0.99	0.91-1.09	0.900
**Median Household Income Quartile of Patient Zip Code**			
<25%	REF	REF	
25-50%	1.03	0.99-1.07	0.178
50-75%	1.06	1.01-1.11	0.009
>75%	1.02	0.97-1.08	0.467
**Emergent Surgery**	9.93	9.20-10.71	<0.001
**Comorbidities**			

© 2021 Brown CS et al. *JAMA Network Open.*

Acute Myocardial Infarction	0.75	0.72-0.78	<0.001
Congestive Heart Failure	1.27	1.23-1.32	<0.001
Peripheral Vascular Disease	1.03	0.99-1.07	0.105
Cerebrovascular Disease	1.37	1.30-1.43	<0.001
Dementia	0.78	0.73-0.83	<0.001
Chronic Obstructive Pulmonary Disease	1.18	1.14-1.21	<0.001
Rheumatoid Disease	1.16	1.10-1.23	<0.001
Peptic Ulcer Disease	1.48	1.34-1.63	<0.001
Mild Liver Disease	1.07	1.00-1.14	0.065
Diabetes	1.18	1.15-1.21	<0.001
Diabetes with Complications	1.17	1.12-1.22	<0.001
Hemiplegia or Paraplegia	0.95	0.85-1.07	0.396
Chronic Kidney Disease	1.24	1.20-1.29	<0.001
Cancer	1.20	1.14-1.26	<0.001
Moderate to Severe Liver Disease	1.56	1.33-1.82	<0.001
Metastatic Cancer	1.53	1.43-1.64	<0.001
Acquired Immunodeficiency Syndrome	1.22	0.98-1.52	0.077

*Effect of primary payer stratified by age < or ≥ 65 years was estimated with a single model including an interaction term between the covariate for primary payer and a categorial variable for age ≥ 65 years
Abbreviations: aOR, adjusted odds ratio; CI, confidence interval; LOS, length of stay

© 2021 Brown CS et al. *JAMA Network Open.*
